# Outcomes of Patients 65 Years or Older After Out-of-Hospital Cardiac Arrest Based on Location of Cardiac Arrest in Japan

**DOI:** 10.1001/jamanetworkopen.2019.1011

**Published:** 2019-03-29

**Authors:** Satoe Okabayashi, Tasuku Matsuyama, Tetsuhisa Kitamura, Kosuke Kiyohara, Takeyuki Kiguchi, Chika Nishiyama, Daisuke Kobayashi, Tomonari Shimamoto, Junya Sado, Takashi Kawamura, Taku Iwami

**Affiliations:** 1 Kyoto University Health Services, Kyoto, Japan; 2 Department of Emergency Medicine, Kyoto Prefectural University of Medicine, Kyoto, Japan; 3 Division of Environmental Medicine and Population Sciences, Department of Social and Environmental Medicine, Osaka University Graduate School of Medicine, Suita, Japan; 4 Department of Food Science, Faculty of Home Economics, Otsuma Women’s University, Tokyo, Japan; 5 Department of Critical Care Nursing, Kyoto University Graduate School of Human Health Science, Kyoto, Japan; 6 Department of Health and Sport Sciences, Osaka University Graduate School of Medicine, Osaka, Japan

## Abstract

**Importance:**

Out-of-hospital cardiac arrest (OHCA) is a major public health issue, and in recent years, the number of OHCAs among the elderly population, aged 65 years or older, has significantly increased in developed countries.

**Objective:**

To evaluate the demographic and clinical characteristics and outcomes of patients 65 years or older who experienced OHCA based on the location—public, residential, or nursing home—where it occurred in Japan.

**Design, Setting, and Participants:**

This prospective, nationwide, population-based cohort study used information collected by the All-Japan Utstein Registry to examine data from 293 615 patients 65 years or older who experienced OHCA during the period from January 2013 to December 2015 in Japan. Data analyses were conducted from June to July 2018.

**Main Outcomes and Measures:**

The primary outcome was 1-month survival with a favorable outcome that was defined as a cerebral performance category score of 1 or 2 (1, good cerebral performance; 2, moderate cerebral disability; 3, severe cerebral disability; 4, coma or vegetative state; and 5, death or brain death). Multivariable logistic regression analyses were conducted to examine favorable outcome by location.

**Results:**

A total of 233 511 patients with OHCA were included in the final analysis; 29 911 (12.8%) occurred in a public location, 157 087 (67.3%) at a residential location, and 46 513 (19.9%) at a nursing home. The median age of the patients was 83.0 years (interquartile range, 76.0-88.0 years), and the proportion of men was 53.1% (124 108 of 233 511). The proportion of favorable neurologic outcomes was 4.5% (1351 of 29 911) in public locations, 1.0% (1555 of 157 087) in residential locations, and 0.6% (301 of 46 513) in nursing homes. Patients with cardiac arrests in public locations had a significantly higher likelihood of achieving a favorable neurologic outcome than those in residential locations (adjusted odds ratio, 1.36; 95% CI, 1.25-1.48), whereas those in nursing homes were less likely to achieve a favorable neurologic outcome (adjusted odds ratio, 0.62; 95% CI, 0.54-0.72). However, this difference in outcomes among patients based on location decreased with age.

**Conclusions and Relevance:**

The outcomes of patients 65 years or older after OHCA differed by the location of the cardiac arrest. These outcomes may be improved by updating existing response measures across all locations.

## Key Points

**Question:**

Do the characteristics and outcomes of out-of-hospital cardiac arrest among patients aged 65 years or older differ by the location where the arrest occurs (public, residential, or nursing home) in Japan?

**Findings:**

In this Japanese nationwide, population-based cohort study of 233 511 persons 65 years or older, the incidence of and the demographic and clinical characteristics of the patients and their outcomes following out-of-hospital cardiac arrest differed according to the location of the arrest. However, the differences in outcomes among patients based on location decreased with age.

**Meaning:**

These results support implementing improved resuscitation strategies for persons experiencing out-of-hospital cardiac arrest in other industrialized countries.

## Teaser

This nationwide, population-based cohort study uses data from the All-Japan Utstein Registry to evaluate the demographic and clinical characteristics and the outcomes of patients aged 65 years or older who experienced out-of-hospital cardiac arrest based on the location—public, residential, or nursing home—where it occurred in Japan.

## Introduction

Out-of-hospital cardiac arrest (OHCA) is a major public health issue in developed countries, and in 2017, approximately 120 000 OHCA events occurred in Japan.^1^ Patients 65 years of age or older accounted for more than 80% of all patients with OHCA in Japan.^1,2^ The number of elderly people in Japan has been rapidly increasing, and the elderly population is predicted to be 30% of the total Japanese population by 2025.^3^ As this population ages, OHCA events are expected to increase. This is a time-sensitive issue that will benefit from understanding OHCA among the elderly population. The long-term, nationwide incidence of elderly persons experiencing OHCA in Japan has been previously reported and is a recognized trend.^2^ The number of elderly people who experience OHCA has increased annually; however, a significant improvement in the proportion of favorable neurologic outcomes has also been noted.

The activities of daily living and functional status vary greatly among the elderly population. Their dwelling places also vary, with their selection sometimes dependent on their degree of independence. Previous studies have demonstrated that the characteristics and outcomes of OHCA differ by location of arrest and by patient age.^4,5,6^ However, few reports have evaluated OHCA regional survival trends by location of arrest among elderly persons.^7,8^ To improve resuscitation strategies for this large population, it is important to obtain updated and comprehensive information about the arrest location.

The All-Japan Utstein Registry is a prospective, nationwide, population-based registry of all patients with emergency medicine service (EMS) personnel–treated OHCA. Our study enrolled approximately 233 000 patients aged 65 years or older who experienced an OHCA of medical origin from January 2013 through December 2015. Using this registry, we aimed to elucidate the nationwide characteristics and outcomes of OHCA among the elderly population based on the location of the cardiac arrest in Japan.

## Methods

### Study Design, Population, and Setting

The All-Japan Utstein Registry maintained by the Fire and Disaster Management Agency (FDMA) is a prospective, nationwide OHCA registry that collects data according to the international Utstein style.^9,10^ The registry methods have been previously described.^11^ The present study included patients 65 years of age or older with OHCA of medical origin occurring in a public location, residential location, or nursing home who were resuscitated by bystanders or EMS personnel and then transported to medical institutions from January 1, 2013, through December 31, 2015. We excluded patients with OHCA whose arrest was witnessed by EMS personnel and whose first documented rhythm was unknown; OHCA at health care facilities or unknown locations were also excluded. This study was approved by the Ethics Committee of Kyoto Prefectural University of Medicine and Osaka University Graduate School of Medicine. Personal identifiers were removed from the database by EMS personnel prior to this study; therefore, the requirement of obtaining written informed consent from each patient was waived by the ethics committees.

Cardiac arrest was defined as the cessation of cardiac mechanical activity as confirmed by the absence of circulation signs.^10^ The cause of arrest was presumed to be of medical origin unless it originated from trauma, drug overdose, drowning, electrocution, or asphyxia based on the current Utstein-style template.^10^ Causes were clinically diagnosed by the physician in charge in collaboration with EMS personnel. This study followed the Strengthening the Reporting of Observational Studies in Epidemiology (STROBE) guideline. Data analysis was conducted from June to July 2018.

### EMS Systems in Japan

Japan had a population of approximately 127 million people in 2015, a geographic area of approximately 378 000 km^2^ with about 750 fire stations equipped with dispatch centers that provided emergency services 24 hours a day.^1^ Emergency life-saving technicians (ELSTs) are highly trained emergency care professionals, and they are qualified to insert an intravenous catheter and an adjunct airway and to use semiautomated external defibrillators (AEDs) for patients with OHCA. Specially trained ELSTs are permitted to intubate patients and administer epinephrine intravenously. Each ambulance has a crew of 3 emergency care personnel, including at least 1 ELST. The EMS personnel are not permitted to terminate resuscitation in the field, except for patients with decapitation, incineration, decomposition, rigor mortis, or dependent cyanosis; therefore, almost all patients with OHCA treated by EMS personnel are transported to a hospital and enrolled in this registry.

### Data Collection and Quality Control

The following resuscitation-related data were collected prospectively: sex, age, etiology of arrest, bystander cardiopulmonary resuscitation CPR, use of public-access AED, dispatcher instruction, first documented rhythm, resuscitation time course, intravascular fluid administration, epinephrine administration, advanced airway management, prehospital return of spontaneous circulation, 1-month survival, and neurologic status 1 month after the event. The first documented rhythm was registered as ventricular fibrillation/pulseless ventricular tachycardia (VF/VT) when bystanders used a public-access AED and provided shocks. The FDMA, in addition to gathering international Utstein-style data,^9^ has been collecting detailed information about the location of arrests since January 2013. The current Utstein-style template categorizes the location of arrest as homes/residences, public areas, workplaces, recreational/sports event areas, streets/highways, health care facilities (clinic/nursing home), educational institutions, and others.^10^ In the present study, these locations were reclassified as residential location (homes/residences), public location (public areas, workplaces, recreational/sports event areas, streets/highways, educational institutions, and others), and nursing homes. The data form was completed by EMS personnel in cooperation with the physician in charge of the patient; these data were integrated into the registry system on the FDMA database server. The data were logic-checked by the computer system and confirmed by the FDMA; incomplete data were returned by the FDMA to the fire station and made complete there. The EMS professional in charge followed up all survivors for 1 month after the event. The physician responsible for the care of the patient evaluated neurologic outcome during a follow-up interview 1 month after successful resuscitation using the cerebral performance category scale: category 1, good cerebral performance; category 2, moderate cerebral disability; category 3, severe cerebral disability; category 4, coma or vegetative state; and category 5, death/brain death.^12^

### Outcome Measures

The primary outcome was 1-month survival with a favorable outcome defined as a cerebral performance category score of 1 or 2.^12^ The secondary outcomes were 1-month survival and prehospital return of spontaneous circulation.

### Statistical Analysis

Patient and EMS characteristics and patient outcomes were stratified by the location of arrest (residential, public, and nursing home). We performed Wilcoxon rank sum tests for continuous variables and χ^2^ tests for categorical variables to assess the differences in patient and EMS characteristics and in patient outcomes according to the location of arrest. For the primary outcome, we then visually described the nonlinear relationship between age and the estimated probability of a favorable neurologic outcome, which was stratified by the location of arrest using predicted margins in the univariable logistic regression model. To assess the contribution of arrest location to favorable neurologic outcome, univariable and multivariable logistic regression analyses were used; odds ratios (ORs), adjusted odds ratios (AORs), and 95% confidence intervals (CIs) were calculated. We selected the following potential confounders that were biologically essential and considered to be associated with clinical outcomes: sex, age category (young-old, 65-74 years; old-old, 75-84 years; oldest-old, ≥85 years), witness status (no, yes), bystander cardiopulmonary resuscitation (CPR) provision (no, yes), shocked using a public-access AED (no, yes), etiology of arrest (cardiac, noncardiac), first documented rhythm (VF/VT, pulseless electrical activity [PEA], or asystole), and EMS response time. All statistical analyses were performed using SPSS, version 25.0J (IBM Corp) and Stata, version 13.0 MP (StataCorp LP). A 2-tailed *P* < .05 was considered statistically significant.

## Results

**Figure 1.**
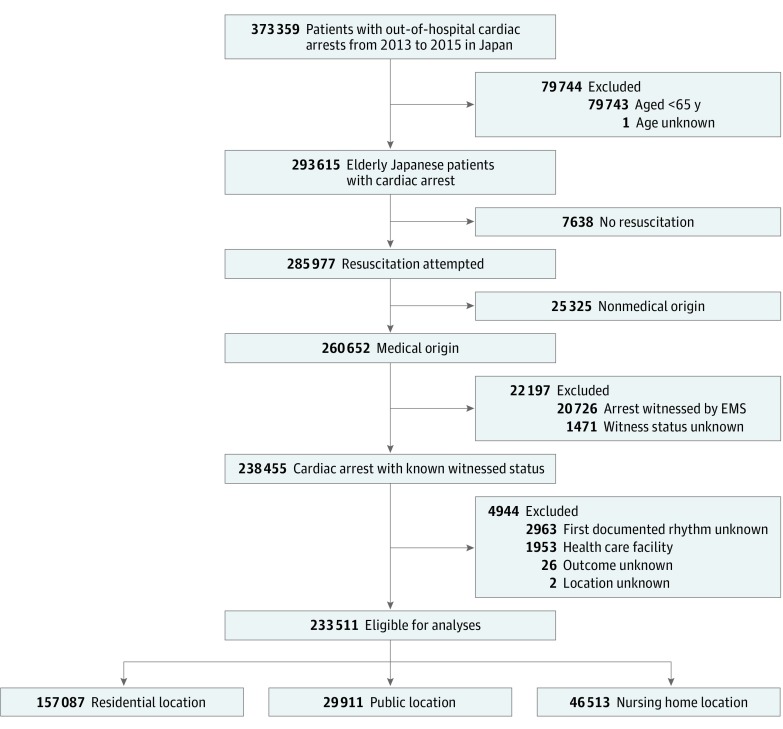
Patient Flow in This Study EMS indicates emergency medical service.

Figure 1 shows the patient flow of this study. During the study period, 373 359 OHCA cases were registered, and 293 615 patients 65 years or older were documented. We excluded 7638 patients who were not resuscitated, 25 325 arrest cases with a nonmedical origin, 22 197 arrest cases witnessed by EMS or whose witness status was unknown, and 4944 cases that met other exclusion criteria. In total, 233 511 patients were included in our final analyses. Among the cardiac arrests meeting inclusion criteria, 29 911 (12.8%) occurred in a public location, 157 087 (67.3%) at a residential location, and 46 513 (19.9%) at a nursing home. The median age of the included patients was 83.0 years (interquartile range, 76.0-88.0 years), and the proportion of men was 53.1% (124 108 of 233 511).

Table 1 presents the patient and EMS characteristics of patient arrests meeting inclusion criteria stratified by the location of arrest. Patients in public locations were more likely than those in residential or nursing home locations to be male, younger, have a cardiac arrest etiology, be witnessed, have a shockable first documented rhythm, and be shocked by public-access AEDs. By contrast, patients in nursing homes were more likely than those in public or residential locations to be women, older, have a noncardiac arrest etiology, and to receive bystander CPR.

**Table 1.** Characteristics of Patients After Out-of-Hospital Cardiac Arrest Stratified by Location of the Cardiac Arrest

Characteristic	Patients, No. (%)			*P* Value^a^
	Residential (n = 157 087)	Public (n = 29 911)	Nursing Home (n = 46 513)	
Men	88 010 (56.0)	19 538 (65.3)	16 560 (35.6)	<.001
Age, median (IQR), y	82 (75-87)	78 (71-85)	87 (82-92)	<.001
Age category				
Young-old (65-74 y)	35 000 (22.3)	10 924 (36.5)	3294 (7.1)	<.001
Old-old (75-84 y)	63 451 (40.4)	11 174 (37.4)	12 948 (27.8)	
Oldest-old (≥85 y)	58 636 (37.3)	7813 (26.1)	30 271 (65.1)	
Etiology of arrest				
Noncardiac	45 064 (28.7)	8109 (27.1)	15 753 (33.9)	<.001
Cardiac	112 023 (71.3)	21 802 (72.9)	30 760 (66.1)	
Bystander				
Witness	53 264 (33.9)	13 352 (44.6)	19 577 (42.1)	<.001
CPR	71 061 (45.2)	14 821 (49.6)	36 725 (79.0)	
First documented rhythm				
VF/VT	7612 (4.8)	6359 (21.3)	2231 (4.8)	<.001
PEA	31 539 (20.1)	7311 (24.4)	10 771 (23.2)	
Asystole	117 936 (75.1)	16 241 (54.3)	33 511 (72.0)	
Shocked by a public-access AED	130 (0.1)	1200 (4.0)	1359 (2.9)	<.001
Dispatcher instruction	100 628 (64.1)	14 062 (47.0)	26 774 (57.6)	<.001
Intravenous fluid	53 594 (40.3)	9371 (34.2)	12 166 (31.3)	<.001
Epinephrine	27 995 (17.8)	6063 (20.3)	7183 (15.4)	<.001
Advanced airway management	137 950 (87.8)	23 557 (78.8)	40 560 (87.2)	<.001
EMS resuscitation times, median (IQR), min				
EMS response time (call to contact with patient)	9 (7-11)	8 (6-10)	9 (7-10)	<.001
Hospital arrival time (call to hospital arrival)	31 (25-38)	30 (24-38)	30 (24-37)	

Abbreviations: AED, automated external defibrillator; CPR, cardiopulmonary resuscitation; EMS, emergency medical services; IQR, interquartile range; PEA, pulseless electric activity; VF, ventricular fibrillation; VT, pulseless ventricular tachycardia.

^a^ Comparisons among the 3 groups were evaluated using Kruskal-Wallis tests for continuous variables and χ^2^ tests or Fisher exact tests for categorical variables.

**Figure 2.**
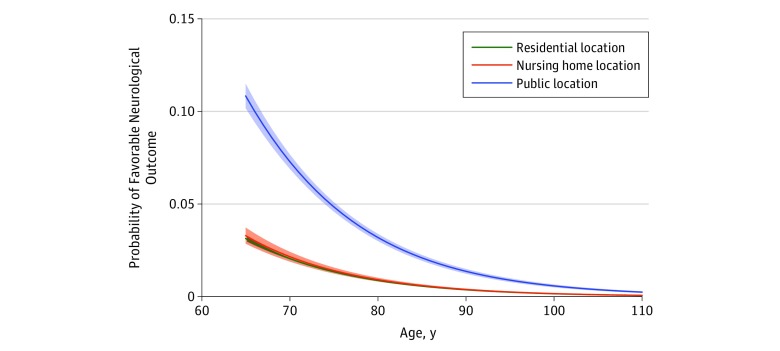
Probability of Favorable Neurologic Outcome After Out-of-Hospital Cardiac Arrest by Age and Location Lines represent predicted values; colored areas, 95% CIs.

The proportions of favorable neurologic outcome were 4.5% (1351 of 29 911) in public locations, 1.0% (1555 of 157 087) in residential locations, and 0.6% (301 of 46 513) in nursing homes (*P* < .001) (Table 2). In the predicted margins, the unadjusted probability of favorable neurologic outcome was generally high in public locations, but the extent of differences among each group decreased with increasing age (Figure 2). Regarding secondary outcomes, patients arresting in public locations also achieved the highest proportion of prehospital return of spontaneous circulation (11.9% vs 6.8% for residential locations and 7.3% for nursing homes; *P* < .001) and 1-month survival (7.9% vs 2.8% for residential locations and 2.6% for nursing homes; *P* < .001) (Table 2).

**Table 2.** Outcomes of Patients After Out-of-Hospital Cardiac Arrest Stratified by Location of the Cardiac Arrest

Outcome	Patients, No. (%)			*P* Value^a^
	Residential (n = 157 087)	Public (n = 29 911)	Nursing Home (n = 46 513)	
Prehospital ROSC	10 656 (6.8)	3545 (11.9)	3379 (7.3)	<.001
1-mo Survival	4379 (2.8)	2373 (7.9)	1226 (2.6)	<.001
CPC 1 or 2	1555 (1.0)	1351 (4.5)	301 (0.6)	<.001

Abbreviations: CPC, cerebral performance category; ROSC, return of spontaneous circulation.

^a^ Comparisons among the 3 groups were evaluated using χ^2^ tests.

In the multivariable logistic regression analysis, patients with OHCAs occurring in public locations had a higher likelihood of achieving favorable neurologic outcomes than those in residential locations (AOR, 1.36; 95% CI, 1.25-1.48), whereas those in nursing homes were less likely to achieve favorable neurologic outcomes (AOR, 0.62; 95% CI, 0.54-0.72). The shockable first documented rhythm at the scene (VF/VT: AOR, 34.99; 95% CI, 30.31-40.40; PEA: AOR, 7.74; 95% CI, 6.69-8.95), bystander witness (AOR, 3.22; 95% CI, 2.93-3.54), bystander CPR (AOR, 1.26; 95% CI, 1.16-1.36), shocked by public-access AEDs (AOR, 2.49; 95% CI, 2.19-2.84), and cardiac arrest etiology (AOR, 1.29; 95% CI, 1.17-1.43) were positively associated with favorable neurologic outcomes (Table 3).

**Table 3.** Factors Associated With 1-Month Survival With Favorable Neurologic Outcome After Out-of-Hospital Cardiac Arrest

Variable	Total No. of Patients	CPC 1 or 2, No. (%)	Crude OR (95% CI)	Adjusted OR (95% CI)
Age category				
Young-old (65-74 y)	49 218	1688 (3.4)	1 [Reference]	1 [Reference]
Old-old (75-84 y)	87 573	1036 (1.2)	0.34 (0.31-0.37)	0.54 (0.49-0.59)
Oldest-old (≥85 y)	96 720	483 (0.5)	0.14 (0.13-0.16)	0.31 (0.27-0.34)
Sex				
Men	124 108	2267 (1.8)	2.15 (1.99-2.32)	1.02 (0.94-1.12)
Women	109 403	940 (0.9)	1 [Reference]	1 [Reference]
Location				
Residential	157 087	1555 (1.0)	1 [Reference]	1 [Reference]
Public	29 911	1351 (4.5)	4.73 (4.39-5.10)	1.36 (1.25-1.48)
Nursing home	46 513	301 (0.6)	0.65 (0.58-0.74)	0.62 (0.54-0.72)
Bystander witness				
No	147 318	594 (0.4)	1 [Reference]	1 [Reference]
Yes	86 193	2613 (3.0)	7.72 (7.06-8.45)	3.22 (2.93-3.54)
Bystander CPR				
No	110 904	1226 (1.1)	1 [Reference]	1 [Reference]
Yes	122 607	1981 (1.6)	1.47 (1.37-1.58)	1.26 (1.16-1.36)
Shocked by public-access AEDs				
No	230 822	2673 (1.2)	1 [Reference]	1 [Reference]
Yes	2689	534 (19.9)	21.15 (19.10-23.42)	2.49 (2.19-2.84)
Etiology of arrest				
Noncardiac	68 926	550 (0.8)	1 [Reference]	1 [Reference]
Cardiac	164 585	2657 (1.6)	2.04 (1.86-2.24)	1.29 (1.17-1.43)
First documented rhythm				
VF/VT	16 202	2044 (12.6)	97.47 (85.34-111.33)	34.99 (30.31-40.40)
PEA	49 621	915 (1.8)	12.68 (11.02-14.60)	7.74 (6.69-8.95)
Asystole	167 688	248 (0.1)	1 [Reference]	1 [Reference]
EMS response time (for 1-min increments)			0.89 (0.88-0.90)	0.91 (0.90-0.92)

Abbreviations: AED, automated external defibrillator; CPC, cerebral performance category; CPR, cardiopulmonary resuscitation; EMS, emergency medical service; OR, odds ratio; PEA, pulseless electrical activity; VF, ventricular fibrillation; VT, pulseless ventricular tachycardia.

## Discussion

By using the nationwide, population-based, prospective OHCA registry of Japan, the present study provided information about the conditions associated with OHCA among patients aged 65 years or older in each of 3 types of locations. The results showed that the number of arrests and the outcomes of OHCA among the patients aged 65 years or older differed by location of arrest. The OHCA cases most frequently occurred in residential locations. Those occurring in public locations had a higher likelihood of achieving favorable neurologic outcomes. However, the survival rate decreased with age, and the difference in outcome status between each location decreased with age. To our knowledge, this is the first nation-level study to investigate the detailed characteristics and outcomes of OHCA among patients aged 65 years or older that focused on the OHCA location. Our results from the study of a “super-aging” society offers future perspectives to other industrialized countries that are making efforts to implement improved resuscitation strategies for elderly persons experiencing OHCA.

As stated above, OHCAs occurred most frequently in residential locations (67.3%) followed by nursing homes (19.9%). The tendency for the majority of OHCA events to occur in private residential locations is consistent with recent worldwide reports indicating a range from 65% to 83%.^13,14^ The proportion of OHCA events occurring in nursing homes was greater than that reported in Japanese local data from 1999 to 2011, which was approximately 12%.^7^ An increasing proportion of OHCA events in nursing homes was reported by a nationwide study in Denmark^15^ and may be attributable to the population composition in developed countries. In Japan, the number of nursing home residents has been increasing since 2011,^16^ and this change may have contributed to our results. The observation of OHCA events among the elderly patients at all locations, including nursing homes, should continue because the number of nursing home residents is expected to continue increasing.^17^

Among the patients in the present study, neurologic outcomes and survival after OHCA events occurring in public locations were the most favorable, which is consistent with findings of previous studies.^7,8^ The younger and healthier backgrounds of these patients combined with better access to AEDs in public locations are likely reasons for the better outcomes.^11,18,19,20^ The present study also showed that the observed difference in outcome was notable in younger patients, but it decreased with age. To our knowledge, this is the first report to identify an age-dependent difference in outcome by location, which suggests the importance of focusing on rescuing younger patients in each location. Moreover, to further improve the outcomes in public locations, it is important to assess the contribution of infrastructure, such as EMS accessibility and AED availability, and the relative density of urban areas in Japan, although we could not obtain this information from our registry. Such information would be helpful for more effective AED installation and the development of a better EMS system.

The present study showed that OHCA outcomes in residential locations were worse than those in public locations even though more OHCAs occurred in residential locations, which has been reported in previous studies.^20^ In addition to lower AED accessibility, residential locations have unique circumstances, such as the patients are more isolated, the OHCA is less likely to be witnessed by a bystander, and a close relationship between patients and their housemates can cause the potential helper to panic.^21^ To solve these problems, it has been suggested to provide a telephone CPR system to guide bystanders^21^ or a mobile-phone positioning system to dispatch CPR-trained laypersons^22,23^ and to use a social network system to address isolation issues.^23^ However, elderly people are not always familiar with mobile phone and social network system technologies, and the number of elderly people living alone will continue to increase.^16^ Therefore, new approaches to avoid isolation will be needed, such as the creation of a watch system for the elderly people within their local community^24^ and a remote OHCA system that uses, for example, wearable devices or other internet technologies.^25^

Despite an increased number of patients receiving bystander CPR and the more frequent use of AEDs compared with those in residential locations, the outcome after OHCA in nursing homes was the worst. This finding was consistent with that of a previous study in Denmark.^15^ Such results have been explained by numerous factors, including older age, increased comorbidity, and overall worse health of the elderly people residing in nursing homes.^26,27,28,29^ The present study showed that 79.0% of patients aged 65 years or older who experienced an OHCA in a nursing home received bystander CPR. However, according to a Japanese survey in 2014, less than 10% of the elderly patients preferred life-prolonging treatment.^16^ Therefore, our results suggest that patients who experience an OHCA in a nursing home may receive bystander CPR against their will. The preferences for end-of-life care differ according to age, race, culture, or socioeconomic status.^30,31^ Therefore, our findings might not be generalizable to other populations. However, to overcome this disparity between patient preference and the current resuscitation system, we have to consider the importance of advanced care planning in nursing homes. Careful and ongoing discussions of end-of-life care in advance with an elderly individual has been reported to increase satisfaction among patients and their families as well as to reduce unnecessary and unwanted resuscitation.^31^ The Ministry of Health, Labour and Welfare of Japan has tried promoting advanced care planning.^32^ As a result, the proportion of individuals who die in nursing homes has been increasing recently.^32^ The number of OHCAs in nursing homes will increase; therefore, it is desirable to more widely spread advanced care planning, including for delivery of CPR in nursing homes.

### Limitations

This study has several limitations. First, the Utstein Registry data does not provide patient risk factors, such as activities of daily living and socioeconomic status before cardiac arrest, that may have influenced the association between location of arrest and patient outcome. Second, this registry does not include the prearrest clinical status, including the prearrest cerebral performance category, and the difference in prearrest cerebral performance category by location of arrest may have contributed to the observed outcomes. Third, we could not address the treatment of each patient after their hospital arrival. Fouth, as with all epidemiologic studies, data integrity and validity and ascertainment bias are potential limitations. However, the population-based study, the use of uniform data collection in accordance with the Utstein-style guidelines for reporting cardiac arrest, and the large sample size may have minimized these potential sources of bias.

## Conclusions

By using the nationwide OHCA registry in Japan, a country with a rapidly aging population, our study showed that the number and characteristics of patients aged 65 years or older with OHCA and their neurologic outcomes differed by the location of their cardiac arrest. Different and necessary measures in each location type are required to improve the outcomes of OHCA among elderly patients. This study provides important information for other industrialized countries interested in implementing improved resuscitation strategies for persons experiencing OHCA.
